# Alveolotomy

**Published:** 1892-02

**Authors:** G. S. Junkerman

**Affiliations:** Cincinnati, Ohio.


ARTICLE IV.

### ALVEOLOTOMY.

BY G. S. JUNKERMAN, M. D., D. D. S., CINCINNATI, OHIO.

Alveolotomy, from whatever cause, means simply perforation of the alveolar process. There may be one, two, three, four, or more perforations, and it would then be styled single, double, triple, quadruple, etc., according to the number made. The alveolotomy may arise in the natural process of disease in nature's effort to throw it off, or from the interference of the surgeon. Alveolotomy, when the result of the surgeon's interference, has perhaps always been performed for the relief of alveolar abscess. More skill is required in performing an alveolotomy than in lancing any other kind of abscess. Abscesses usually manifest the point at which they wish to make their exits; the point for alveolotomy must be determined by the surgeon himself.

Alveolotomy may be performed upon any of the alveolar cavities of the thirty-two teeth; but any other than a single operation ceases to be practical; therefore that can best be performed on any one of the eight anterior teeth of both jaws. Upon these teeth the operation is made with the best results in the order named,—the lateral incisors, the bicuspids, the central incisors, and the canines. The operation is more often indicated in the upper than in the lower jaw, and is followed by better results.

The indications for alveolotomy always presuppose the presence of an abscess; but the operation is preferably performed at the termination of the abscess, or during its progress. The end of an abscess, it will be argued, is an alveolotomy performed by nature for the exit of pus. This is true, and nature's operation is the best indication for that of the surgeon. You cannot style that an alveolotomy where the surgeon dilates the opening already made by nature to remove the abscess and disinfect the cavity. It would be unwise to interfere by operation on cases of abscess where nature has not yet made an opening. The operation should be avoided at the abscess stage, as being only a means of inflicting additional pain, and in no manner accelerating the arrival of relief. The true operation of alveolotomy is indicated when drainage is required, or in case of burrowing abscesses where the pus has invaded the soft tissue. The operation is most frequently indicated in abscess of the lateral incisor. So closely related is this operation and the lateral incisor that it might be called *lateral* alveolotomy. The oral surgeon cannot speak of abscesses of the teeth in one category. To the observing operator the difference of lateral abscess from that of any of the other teeth must again and again force itself upon the attention. It would be impossible to give statistics; but in every one's experience there must appear the fact of the frequency of these becoming abscesses of the hard palate. All abscesses of the hard palate arise from the lateral incisor. If your attention is turned to the anatomy of the alveolar process of the superior maxillary bone in the proximity of the lateral incisor, you will observe that the arrangement of the parts is responsible for this frequency of palatine abscess. In cases where the root of the lateral is not extraordinarily long, the wall of bony tissue posteriorly is thinner than anteriorly, or equally as thin as the anterior wall, and consequently the resistance of both walls approach an equality. Therefore the abscess is as likely to make its exit posteriorly as anteriorly. With the central and the canine the case is different. The roots of these teeth extend further into the cancellous tissue, and the posterior walls become correspondingly thicker than the anterior. Knowing this anatomical fact, it only becomes a problem of physics to determine why these lateral abscesses result so frequently in those of the hard palate. If abscess of the hard palate be present, no matter how diverse the indications may be, it will be wise to always suspect the lateral incisor. This tooth furnishes the best case for an alveolotomy. Where the abscess has extended to the hard palate, opening it in this position does not furnish sufficient drainage. There is too much cancellous issue and too great an extent of surface to drain by one opening. These are then the circumstances which indicate the perforation of the alveolus, by which a perfect drainage can be secured. The alveolotomy may be performed almost painlessly by the previous injection of cocaine. The perforation should be made at the apex of the lateral or as near to the apex as possible. A spear-shaped bur with rapid rotations of the engine should be used to perform the operation. Experience and the acquirement of skill are the only guides to discovering the exact position for the perforation. To sum up, then, the object of this paper has been to identify with surgery the operations which I think I have properly designated alveolotomy.—*International Dental Journal.*

